# Supplementary material for: Quantitative single-cell live imaging links HES5 dynamics with cell-state and fate in murine neurogenesis
Source: Nat Commun. 2019 Jun 27;10:2835. doi: 10.1038/s41467-019-10734-8 (PMC6597611; doi:10.1038/s41467-019-10734-8)
Supplement: Supplementary file 5 — Reporting Summary [file 41467_2019_10734_MOESM5_ESM.pdf]

nature research

Corresponding author(s): Cerys Manning and Nancy Papalopulu

Reporting Summary

Nature Research wishes to improve the reproducibility of the work that we publish. This form provides structure for consistency and transparency in reporting. For further information on Nature Research policies, see Authors & Referees and the Editorial Policy Checklist.

Statistical parameters

When statistical analyses are reported, confirm that the following items are present in the relevant location (e.g. figure legend, table legend, main text, or Methods section).

n/a	Confirmed	
☐	☒	The exact sample size (*n*) for each experimental group/condition, given as a discrete number and unit of measurement
☐	☒	An indication of whether measurements were taken from distinct samples or whether the same sample was measured repeatedly
☐	☒	The statistical test(s) used AND whether they are one- or two-sided<br>*Only common tests should be described solely by name; describe more complex techniques in the Methods section.*
☐	☒	A description of all covariates tested
☐	☒	A description of any assumptions or corrections, such as tests of normality and adjustment for multiple comparisons
☐	☒	A full description of the statistics including central tendency (e.g. means) or other basic estimates (e.g. regression coefficient) AND variation (e.g. standard deviation) or associated estimates of uncertainty (e.g. confidence intervals)
☐	☒	For null hypothesis testing, the test statistic (e.g. *F*, *t*, *r*) with confidence intervals, effect sizes, degrees of freedom and *P* value noted<br>*Give P values as exact values whenever suitable.*
☐	☒	For Bayesian analysis, information on the choice of priors and Markov chain Monte Carlo settings
☐	☒	For hierarchical and complex designs, identification of the appropriate level for tests and full reporting of outcomes
☐	☒	Estimates of effect sizes (e.g. Cohen's *d*, Pearson's *r*), indicating how they were calculated
☐	☒	Clearly defined error bars<br>*State explicitly what error bars represent (e.g. SD, SE, CI)*

Our web collection on statistics for biologists may be useful.

Software and code

Policy information about availability of computer code

Data collection	Zen2.1 was used for image, movie and FCS data acquisition on a LSM880 Carl Zeiss microscope. Imaris v9.1.2 was used for tracking single neural progenitor cells in 3D movies of E10.5 spinal cord tissue. ImageJ 1.43 was used for image analysis and manual segmentation.
Data analysis	R Studio and GraphPad Prism 7/8 were used for statistical analysis. Data fitting for detections of oscillations has been implemented in Matlab R2015a using the GPML toolbox (Rassmussen and Hannes 2010) and custom designed routines available at http://gaussianprocess.org/gpml/code/matlab/doc/. Code for stochastic model of genetic auto-repression and Bayesian inference available online under https://github.com/kursawe/hesdynamics. Custom designed Matlab (R2015a) routines for analysis of FCS available on request.

For manuscripts utilizing custom algorithms or software that are central to the research but not yet described in published literature, software must be made available to editors/reviewers upon request. We strongly encourage code deposition in a community repository (e.g. GitHub). See the Nature Research guidelines for submitting code & software for further information.

Data

Policy information about availability of data

All manuscripts must include a data availability statement. This statement should provide the following information, where applicable:

- Accession codes, unique identifiers, or web links for publicly available datasets
- A list of figures that have associated raw data
- A description of any restrictions on data availability

No large datasets generated in this study. However we have provided raw data for figures in the Source Data file and links to raw imaging files in the Data availability statement. We have also included -
- nuclear Venus::HES5 concentrations from fluorescence correlation spectroscopy. Raw counts available on request from the corresponding authors.
- Single-cell Venus::HES5 intensity, H2B::mCherry intensity and positional information extracted from live imaging of untreated, DMSO and DBZ Notch inhibitor treated tissue.

Field-specific reporting

Please select the best fit for your research. If you are not sure, read the appropriate sections before making your selection.

☒ Life sciences ☐ Behavioural & social sciences ☐ Ecological, evolutionary & environmental sciences

For a reference copy of the document with all sections, see nature.com/authors/policies/ReportingSummary-flat.pdf

Life sciences study design

All studies must disclose on these points even when the disclosure is negative.

Sample size	No sample-size calculations were performed.
Data exclusions	Data exclusion was not pre-determined. For movie tracking only cells that started the movie in Venus::HES5 positive domain were analysed as these would be the Sox2+ progenitors and we were interested in tracking progenitors. In Fig. 6e outliers (2) were removed using the ROUT methods.
Replication	Multiple independent experiments were performed to verify reproducibility of findings. All attempts at replication were successful. Hierarchical clustering was performed separately for each experiment to ensure reproducibility in clustering.
Randomization	Randomization was not relevant to this study.
Blinding	Blinding was not relevant to the tracking of cells in DMSO vs DBZ treated slices as the DBZ resulted in a global down-regulation of Venus::HES5 and was recognisable. We blinded the analysis of short-term Venus::HES5 dynamics by storing the positional and cluster information of each cell in a meta-data file separate to the file containing data to analyse.

Reporting for specific materials, systems and methods

Materials & experimental systems

n/a	Involved in the study	
☒	☐	Unique biological materials
☐	☒	Antibodies
☐	☒	Eukaryotic cell lines
☒	☐	Palaeontology
☐	☒	Animals and other organisms
☒	☐	Human research participants

Methods

n/a	Involved in the study	
☒	☐	ChIP-seq
☒	☐	Flow cytometry
☒	☐	MRI-based neuroimaging

Antibodies

Antibodies used	RabMAb Anti-HES5 [EPR15578], Abcam, ab194111, Lot GR214854-4<br>Mouse monoclonal anti-alpha-tubulin, clone DM1A, Sigma, T9026<br>Rabbit MAb anti-Beta-III-tubulin (D71G9), Cell Signalling Technology, 5568S, lot 4<br>Rabbit anti-SOX2, Abcam, ab97959, lot GR293487-3<br>Mouse anti-NeuN, clone A60, Merck, MAB377, lot LV1359479

Mouse monoclonal anti-NKX2.2 (74.5A5), supernatent, Developmental Studies Hybridoma Bank
Mouse monoclonal anti-PAX7, supernatent, Developmental Studies Hybridoma Bank
Rabbit polyclonal anti-OLIG2 EMD Millipore, AB9610, lot 3109748
Mouse monoclonal anti Isl1/2, clone 39.4D5, supernatent, Developmental Studies Hybridoma Bank

Validation

RabMAb Anti-HES5 - Abpromise guarantee for use against mouse HES5 in western blot.
Mouse monoclonal anti-alpha-tubulin - Sigma report - The antibody is specific for α-tubulin in immunoblotting assays
Rabbit MAb anti-Beta-III-tubulin (D71G9) - Cell Signalling Technology report β3-Tubulin (D71G9) XP® Rabbit mAb detects endogenous levels of total β3-tubulin protein. This antibody does not cross-react with tubulin isoforms expressed in non-neuronal cells.
Rabbit anti-SOX2 - Abpromise guarantee for use against mouse SOX2 in immunohistochemistry - frozen
Mouse anti-NeuN, clone A60 - Merck 100% performance guarantee against mouse NeuN in immunohistochemistry
Mouse monoclonal anti-NKX2.2 (74.5A5) - deposited to the DSHB by Jessell, T.M. / Brenner-Morton, S. Tested positive for reactivity with mouse.
Mouse monoclonal anti-PAX7 - deposited to the DSHB by Kawakami, A. Tested positive for reactivity with mouse.

Eukaryotic cell lines

Policy information about cell lines

Cell line source(s)

NS-E cells were a gift from Jennifer Nichols (Cambridge Stem Cell Institute, UK).

Authentication

No authentication performed.

Mycoplasma contamination

NS-E cells tested negative for mycoplasma.

Commonly misidentified lines (See ICLAC register)

Name any commonly misidentified cell lines used in the study and provide a rationale for their use.

Animals and other organisms

Policy information about studies involving animals; ARRIVE guidelines recommended for reporting animal research

Laboratory animals

Venus::HES5 knock-in mice (ICR.Cg-Hes5<tm1(venus)Imayo>)(Imayoshi et al. 2013) were obtained from Riken Biological Resource Centre, Japan and maintained as a homozygous line.
Sox1Cre:ERT2 mice (Sox1tm3(cre/ERT2)Vep (Kicheva et al. 2014) were obtained from James Briscoe with the permission of Robin Lovell-Badge.
R26R-H2B::mCherry mice (Abe et al. 2011) were obtained as frozen embryos from Riken Centre for Life Science Technologies, Japan and C57Bl6 mice were used as surrogates.
Triple transgenic embryos were generated and used at E10.5, sex of embryos used unknown.

Wild animals

Provide details on animals observed in or captured in the field; report species, sex and age where possible. Describe how animals were caught and transported and what happened to captive animals after the study (if killed, explain why and describe method; if released, say where and when) OR state that the study did not involve wild animals.

Field-collected samples

For laboratory work with field-collected samples, describe all relevant parameters such as housing, maintenance, temperature, photoperiod and end-of-experiment protocol OR state that the study did not involve samples collected from the field.
