# Supplementary material for: Quantitative single-cell live imaging links HES5 dynamics with cell-state and fate in murine neurogenesis
Source: Nat Commun. 2019 Jun 27;10:2835. doi: 10.1038/s41467-019-10734-8 (PMC6597611; doi:10.1038/s41467-019-10734-8)
Supplement: Supplementary file 6 — Description of Additional Supplementary Files [file 41467_2019_10734_MOESM6_ESM.docx]

**Supplementary Movie Legends**

**Title: Supplementary Movie 1.
Description:** Movie of transverse ex-vivo slice through E10.5 Venus::HES5 Sox1Cre:ERT2 R26R-H2B::mCherry spinal cord with mosaic nuclear labeling induced by low-dose tamoxifen. Example cells from each dynamic expression cluster indicated and their normalized Venus::HES5 intensity shown. Dashed line indicates approximate position of ventricle. Scale bar 30μm. Movie lasts 12 hours, images taken every 15 mins.

**Title: Supplementary Movie 2.
Description:** Movie of transverse ex-vivo slice through E10.5 Venus::HES5 Sox1Cre:ERT2 R26R-H2B::mCherry spinal cord with mosaic nuclear labeling induced by low-dose tamoxifen. Example cell showing transition from noisy to oscillatory Venus::HES5 dynamics. Transition in dynamics coupled to division and subsequent migration away from ventricle. Dashed line indicates approximate position of ventricle. Movie lasts 14.25 hours, images taken every 15 mins.
