# Supplementary material for: Quantitative single-cell live imaging links HES5 dynamics with cell-state and fate in murine neurogenesis
Source: Nat Commun. 2019 Jun 27;10:2835. doi: 10.1038/s41467-019-10734-8 (PMC6597611; doi:10.1038/s41467-019-10734-8)
Supplement: Supplementary file 2 — Peer Review [file 41467_2019_10734_MOESM2_ESM.pdf]

Editorial Note: Parts of this peer review file have been redacted as indicated to remove third-party material where no permission to publish could be obtained.

Reviewers' comments:

Reviewer #1 (Remarks to the Author):

In this manuscript, the authors examined the expression dynamics of Hes5 in neural progenitors by using Venus:Hes5 knock-in mice. They performed live imaging of Venus-Hes5 expression in the developing spinal cord and classified Hes5-expressing cells into four groups, depending on the expression patterns: fluctuating expression in clusters 1 and 2 and decreasing expression in clusters 3 and 4. Cells in clusters 1 and 2 are progenitors while those in clusters 3 and 4 are differentiating cells. Because more cells in clusters 3 and 4 tended to show oscillations than those in clusters 1 and 2, it is likely that progenitors show high and noisy Venus-Hes5 expression while differentiating cells tend to show decreasing but oscillatory Venus-Hes5 expression. Lastly, the authors performed mathematical modeling and suggested that the Hes5 oscillator may operate very close to the bifurcation boundary between aperiodic and oscillatory dynamics.

This is an interesting and well-executed work revealing the dynamic expression patterns of Hes5 in the developing spinal cord. This manuscript would be strengthened after the flowing issues are addressed.

1. The authors found that only 30-40% of dividing progenitors showed oscillatory Venus-Hes5 expression, although previous imaging analysis with a destabilized luciferase reporter showed that Hes5 expression is oscillatory in most cultured neural progenitors (Imayoshi et al. 2013). Hes5 expression dynamics could be different between tissues and dissociated cultures, but it is not clear whether imaging the fluorescent activity of Venus-Hes5 fusion protein represents the Hes5 expression levels. Because Venus is much slower for maturation than luciferase, the authors should be careful about the interpretation of their imaging data. It is possible that imaging the fluorescent activity of Venus may miss the oscillatory expression with two-hour periodicity. This possibility can be tested by dissociation cultures of neural progenitors prepared from the spinal cord of Venus:Hes5 knock-in mice to see whether the fluorescent activity oscillates like luciferase activity. The authors should clarify this issue and discuss the limitation of their imaging method.

2. The functional significance of different Hes5 expression patterns in neural progenitors (clusters 1 and 2) is not clear. The authors should analyze whether neural progenitors show different characteristics, such as cell cycle length, cell cycle phase (both can be estimated by interkinetic nuclear migration), and timing of neuronal differentiation, depending on oscillatory or non-oscillatory, or stable high or stable low Hes5 expression.

3. Related to the above comment (#2), the functional significance of different Hes5 expression patterns in differentiating cells (clusters 3 and 4) is not clear. Is there any difference in differentiating cell types between oscillatory and non-oscillatory, or clusters 3 and 4?

4. Regarding the live imaging method, the authors should describe how many images with what depth intervals along the z axis were taken at each time point. Another issue is that in Supplementary Figure 2d, Venus-Hes5 signals in homozygous are not twice as high as heterozygous samples, suggesting that substantial background fluorescence may be included. The authors should remove the background fluorescence to quantify Venus-Hes5 signals.

--

Reviewer #2 (Remarks to the Author):

In this study by the Papalopulu lab, the authors develop quantitative approaches coupled with live imaging of a Venus::Hes5 reporter to describe the kinetics of HES5 protein expression in the mouse developing spinal cord. HES5 is a canonical target of the Notch pathway and acts as a repressor of

pro-neuronal genes to maintain a progenitor state. Prior studies have shown that targets of the Notch pathway can display oscillatory expression in neural progenitors. These published studies indicate that Notch oscillations promote progenitor maintenance, while sustained expression promotes differentiation. In contrast, one key point of the current manuscript is that both progenitors and differentiating cells can transition between oscillatory and non-oscillatory states for HES5 expression. Further, using non-supervised hierarchical clustering of expression profiles, the authors show that putative differentiating cells are more likely to exhibit HES5 oscillations than putative progenitors. The authors conclude that in differentiating cells, increased likelihood of oscillations is associated with decreased HES5 expression, which coupled together may have a potent impact upon downstream target genes. Finally, using mathematical modeling based on measured Hes5 mRNA and protein turnover, the authors make the compelling point that oscillations arise from noise.

This is a novel and well written study. Using unbiased quantification of gene expression in tissue, this study illuminates fundamental, overlooked and therefore undocumented characteristics of oscillatory gene expression associated with neural differentiation in vivo. It relies upon elegant data using state of the art techniques. Given the potential discordance with previous data from the literature, some additional experiments are needed to support the authors' claims. If the authors can accomplish these experiments, this study will be valuable to the fields of neural differentiation and cell fate decisions at large.

Major concerns

The observation of differential oscillatory expression within progenitors vs differentiating cells relies on indirect clues about cell fate, such as relative distance from the ventricle, distance traveled, and presence of dividing cells. While these are valuable, a direct method is needed to verify the fate of cells as progenitors and neurons, such as using a Dcx-DsRed or Sox2-GFP transgene or immunofluorescence analyses following live-imaging. The authors need to show that oscillations occur in progenitors definitively and that it changes as cells differentiate. It would also be valuable if the authors can use a genetic means to induce more progenitors and show that they all behave like clusters 1 and 2, ie. the reciprocal experiment to DBZ treatment.

Distance from the ventricle could also correlate with cell cycle phase in progenitors. Thymidine analog-based analyses could be performed in order to interrogate this question.

I also have some questions regarding classification of cells. The classification of cluster 3 as differentiating cells is clear from the data included in Figure 3. However the cells of cluster 4 have a similar number of dividing cells as cluster 2 (Figure 3G) and exhibit similar distances from the ventricle (Figures 3C and F). The Notch inhibition experiments result in cells with patterns that are most similar to cluster 3 (comparing Figures 2E and 3K) however the authors conclude that DBZ treatment induces cluster 4 cells. How do the authors reconcile this and is it possible these clusters are overly simplified? Further characterization of these so-called DBZ induced differentiated cells is needed. For example, with the DBZ treatment do the authors see a similar fraction of dividing cells as in Cluster 4 of Figure 2?

Minor points

On page 7 they refer to dynamic fluctuations represented by scenario Fig. 2a ii but it seems these should refer to Fig. 2a i.

Do the authors observe any influence of local neighbor on fluctuations or coordination of oscillations amongst neighboring cells, suggesting a non-cell intrinsic mechanism at play?

In figure 1 they monitor Sox1-Cre recombined cells after several hours, which they refer to as neural

progenitors, but these could be neuronal progeny. This point should be modified.

--
Reviewer #3 (Remarks to the Author):

The manuscript by Manning et al describes an experimental and computational analysis of single cell Hes5 dynamics in the developing mouse spinal cord. The authors use quantitative live imaging of Venus:Hes5 knock-in reporter to look at both the mean fluorescence and the fluctuations around the mean of Hes5 levels in neural progenitors in the spinal cord. The authors identify 4 groups of cells exhibiting different dynamics of the mean fluorescence, where 2 groups (group 1 and 2) correspond to generally constant levels of Hes5 and the two other groups correspond to decay in fluorescence over time (groups 3 and 4). They then show that two latter groups are mostly associated with cells transitioning into a differentiated. The authors then argue that some of the cells exhibit periodic oscillations around the mean (as opposed to aperiodic fluctuations), and that these periodic oscillations occur predominantly the decay in fluorescence in groups 3 and 4. The authors use a stochastic delayed feedback model to explain the observed cellular dynamics. They argue that the periodic fluctuations may be important for the transitions into a differentiated state.

Although the issue of cellular dynamics is indeed an important topic, and although the authors do a very detailed and careful analysis of the Hes5 single cell dynamics, I unfortunately do not find the study to be very compelling, nor the results (particularly those describing the oscillations) very convincing. The main issue is that unlike previous studies in neural progenitors in the brain, the oscillations reported here are very weak (namely, have very low amplitude about the mean). Although I appreciate the quantitative effort to show that a periodic model fits better some of the curves than aperiodic fluctuating model, I am still skeptical whether the observed fluctuations are real oscillations or not (see more detailed concerns below). Furthermore, even if these oscillations are real, it is unclear to me whether they represent an oscillating state of the cell. To really show that the cells have an oscillating state, the authors should have a two color reporter in two loci of the same gene or of two separate genes that show correlated oscillations. I realize this is a big task experimentally, but I think that the authors would have hard time to convince the community that these are oscillatory states without such evidence.

In addition to this main criticism there are several more specific points:

1. The authors show that cells in groups 3 and 4 exhibit a decays in the signal and that this decay correlate with exit from the ventricular zone to the mantle zone. Do any of the H2B:mCherry cells also exhibit such a decay? Is it correlated with distance away from the ventricle?

2. The oscillating are predominantly observed in groups 3 and 4 during the decay. Is there a correlation between mean Hes5 levels and the oscillatory behaviors? Namely, could it be that the observed oscillatory behavior appears when the signal gets weaker?

3. Are the oscillations also observed when DBZ is added? If so, is there a larger fraction of cells exhibiting oscillations with DBZ (one would predict that based on the observation that oscillations appear when Hes5 signal decays).

4. In the paragraph discussing figure 4 (page 9) it is said that 47% of the cells exhibit oscillatory behavior. However, in fig 4c the fraction of oscillating cells seem to be lower in almost all groups tested. Why is there such a discrepancy?

5. Figure 4e shows that clusters 1 and 2 have higher noise than clusters 3 and 4. Could this simply reflect the higher mean levels in these groups (note that the CV of clusters 1 and 2 are lower as showin in fig. 2h).

6. In figure 4f likelihood of oscillatory behavior is shown to be higher for cells away from the ventricle. Could the authors also show how the likelihood depend on absolute mean level of Hes5?

7. Similarly, the authors should also show how the noise in Fig 4g depends on mean level.

8. The authors argue that the typical behavior is that oscillation appear only after the Hes5 signal starts to decay (Figure 4h and 6f). Can the authors determine what fraction of cells exhibit this type of typified behavior? For example, by calculating in what fraction of cells the oscillations appear on the second half of the measurements.

9. I find the stochastic model to be not particularly illuminating. First, it is unclear why the model applies only to clusters 1 and 2 (first sentence in section 7 of the result). Second, it is not clear to me what is gained by the model. The fact that such a model CAN fit partially fluctuating and partially oscillating behaviors is not particularly surprising. Does the model generate a prediction that can be tested experimentally?

Editor's comments
We hope you will find the referees' comments useful as you decide how to proceed. In particular, please include further data to **address the main (serious) points raised by referees 1 and 3 regarding some of your conclusions regarding what the oscillations mean/cell types they identify**. Also, **why periodic oscillations occur predominantly during the decay in fluorescence in groups 3 and 4**. As I am sure you are aware, irrespective of whether new data are required to address a reviewer query, we would expect you to address all points in detail in your response to the reviewers/a revised manuscript (if appropriate).

We have found the reviewer's comments constructive and they helped us to improve the paper by additional experimentation and textual additions/changes. Thank you for giving us the opportunity to respond to these comments. Our detailed response is shown below. Please note that we have copied the new data in our response to make it easier for the reviewers to inspect the data. All new data have also been added to the paper in the places indicated.

Reviewers' comments:

Reviewer #1 (Remarks to the Author):

In this manuscript, the authors examined the expression dynamics of Hes5 in neural progenitors by using Venus:Hes5 knock-in mice. They performed live imaging of Venus-Hes5 expression in the developing spinal cord and classified Hes5-expressing cells into four groups, depending on the expression patterns: fluctuating expression in clusters 1 and 2 and decreasing expression in clusters 3 and 4. Cells in clusters 1 and 2 are progenitors while those in clusters 3 and 4 are differentiating cells. Because more cells in clusters 3 and 4 tended to show oscillations than those in clusters 1 and 2, it is likely that progenitors show high and noisy Venus-Hes5 expression while differentiating cells tend to show decreasing but oscillatory Venus-Hes5 expression. Lastly, the authors performed mathematical modeling and suggested that the Hes5 oscillator may operate very close to the bifurcation boundary between aperiodic and oscillatory dynamics.

This is an interesting and well-executed work revealing the dynamic expression patterns of Hes5 in the developing spinal cord. This manuscript would be strengthened after the flowing issues are addressed.

1. The authors found that only 30-40% of dividing progenitors showed oscillatory Venus-Hes5 expression, although previous imaging analysis with a destabilized luciferase reporter showed that Hes5 expression is oscillatory in most cultured neural progenitors (Imayoshi et al. 2013). Hes5 expression dynamics could be different between tissues and dissociated cultures, but it is not clear whether imaging the fluorescent activity of Venus-Hes5 fusion protein represents the Hes5 expression levels. Because Venus is much slower for maturation than

luciferase, the authors should be careful about the interpretation of their imaging data. It is possible that imaging the fluorescent activity of Venus may miss the oscillatory expression with two-hour periodicity. This possibility can be tested by dissociation cultures of neural progenitors prepared from the spinal cord of Venus:Hes5 knock-in mice to see whether the fluorescent activity oscillates like luciferase activity. The authors should clarify this issue and discuss the limitation of their imaging method.

Unfortunately, it is a misconception that "previous imaging analysis with a destabilized luciferase reporter showed that HES5 expression is oscillatory in most cultured neural progenitors. The paper that the reviewer refers to, does indeed report HES5 dynamics(Imayoshi et al., 2013), but they are showing only one cell of the relevant reporter i.e. of the Venus::HES5 knock-in mouse. As can be seen in Supplementary Figure S9A,B (page 22 of Supplementary Information) of Imayoshi et et al. this paper shows one example each of a neural progenitor from the Eluc-Hes5 knock-in mouse and Venus-Hes5 knock-in mouse (the same mouse as used in this study). The same paper also shows 2 example cells from the pHes5-NLS-Ub-luc2 transgenic mouse in Supplementary Figure S10A,B,C (page 23 of Supplementary Information) where the luc2-HES5 fusion protein is driven by a 3kb region of the Hes5 promoter. This is of course a very different type of a reporter because it is not a knock-in but again there is no analysis at a population level. This published report is helpful in showing that both luciferase and fluorescence based reporters can be used to image HES5 dynamics. However, there is no statistical analysis of the population in the Imayoshi paper to allow one to conclude that "most" progenitors oscillate or what the periodicity would be. We are also not aware that the HES5 period has been reported anywhere else. Thus, a description of dynamics at a population level, backed up by statistical analysis, and a characterisation of the periodicity is reported for the first time in our paper. We believe that this is a major strength of our work.

[Redacted]

This reviewer is also concerned that Venus may not be as faithful in reporting the endogenous dynamics as a luciferase reporter due to the maturation time of the fluorophore. The reviewer is particularly concerned that the use of Venus prevented us from detecting oscillations of 2 hr periodicity. First, we note that the maturation time of Venus is short ($t_{1/2}$ of 15 mins (Ball et al., 2014) compared to HES5 protein half-life (87 mins, Supp Fig.1). Second, as explained above there is no previous report of HES5 periodicity. HES1 has been reported to have a periodicity of 2-5 hrs (depending on the cell type) but HES5 could have longer period because HES5 protein has a longer half-life than HES1 (HES5; 87 minutes, (Supp. Fig 1) HES1, 25 mins (Kobayashi et al., 2015)). Also please note the presence of cells with 2 hour periods within the distribution of periods (Fig.4c) Thirdly, we have taken the reviewers suggestion and we have looked at the dynamic behaviour of HES5 in dissociated cultured spinal cord cells (Supp. Fig 8 and Rebuttal Figure 2 below). We found that there is a significant increase in the number of oscillatory cells in vitro (Rebuttal Figure 2B, 77% dissociated Venus::HES5$^{+/-}$ cells versus 41% in tissue Venus::HES5$^{+/-}$ slices). There is also

an increase in the mean period in dissociated single Venus::HES5$^{+/-}$cells in vitro (Rebuttal Figure 2C, 3.9 hrs dissociated Venus::HES5$^{+/-}$ cells versus 3.3 hrs in tissue Venus::HES5$^{+/-}$ slices), showing that indeed the dissociation of cells alters their dynamics compared to the tissue environment which will be the subject of future investigation. Taken together, these findings give us confidence that our reporter is able to detect oscillations when these take place.

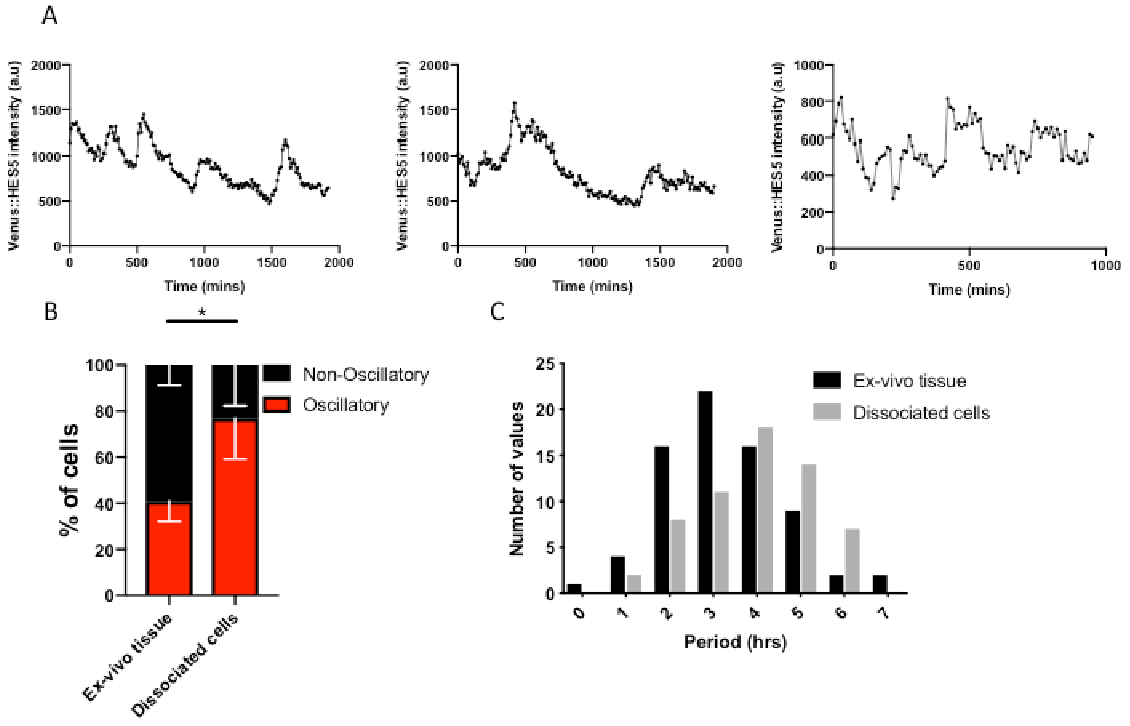

Figure 2 Venus::HES5 expression is oscillatory in dissociated spinal cord primary neural stem cells. A). Example HES5 expression dynamics from single dissociated spinal cord neural progenitor cells cultured in vitro. Comparison of B). percentage of cells with oscillatory Venus::HES5 and C) periods in ex-vivo spinal cord tissue slices versus dissociated cells from spinal cord.

Nevertheless, we agree with a reviewer that a discussion of the different imaging methods would be useful to the reader. Specifically, luciferase imaging requires signal integration of 10-20mins thus underreporting on the dynamics and also lacks a detection pinhole so reduces the spatial resolution needed for tissue environment imaging. Both of these are possible with the fluorescent Venus::HES5 fusion reporter. In addition, the fluorescent knock-in reporter allowed us to undertake quantitative fluorescence correlation spectroscopy (FCS) to measure HES5 concentration in the nucleus, which we have used to parameterise mathematical models.

In conclusion, we are confident that we have used the right reporter and our analysis advanced our understanding of the HES5 dynamics in neural tissue. We have added the new data on Venus::HES5 expression dynamics dissociated cells in Supp.Fig 8 and a discussion on the different imaging reporters on page 9 of the revised manuscript .

2. The functional significance of different Hes5 expression patterns in neural progenitors (clusters 1 and 2) is not clear. The authors should analyze whether neural progenitors show different characteristics, such as cell cycle length, cell cycle phase (both can be estimated by interkinetic nuclear migration), and timing of neuronal differentiation, depending on oscillatory or non-oscillatory, or stable high or stable low Hes5 expression.

There are 2 points to this question, the functional significance in progenitors and correlation with parameters additional to the ones we have used in our paper. We will address these in turn. First, with regards to the dynamic HES5 expression pattern in progenitors (cluster 1 and 2), in the manuscript, we propose that the functional significance is to keep active expression but plastic, that is, poised to undergo a transition. This hypothesis is borne out by experimental observation that shows that less than half of the cells oscillate (Figure 4d) and our computational experimentation which shows that the system is poised at a bifurcation point (Figure 5e,f). It is unlikely that the oscillations in progenitors are decoded as such, because their amplitude is small. However, in the revised manuscript we provide evidence that in differentiated cells (cluster 3 and 4) different dynamics correlate with different neuronal populations (Figure 6a-c), suggesting that oscillations are decoded during the route to differentiation (see reply to next questions for details).

The second point regards any possible correlation of progenitor dynamics with cell cycle, INM and timing of differentiation. We have developed the spinal cord embryonic slice culture method and used it for the first time. Unfortunately, we are limited experimentally by how long we can keep these embryonic slices alive, healthy and in good shape to be able to observe cells long enough to answer these questions (currently about 12 hrs). For example, cell-cycle length at this stage is at least 13 hours (Molina and Pituella, Developmental Biology 2017) therefore, we have not been able to image cells with two divisions.

However we have been to estimate the cell-cycle phase in cells of cluster 1 and 2. As the reviewer implies, cells undergo INM, whereby nuclei undergo S phase further from the ventricle and return to the ventricle for M phase, separated by G1 and G2 phases. We have calculated the cell cycle phases, based on how long nuclei spend is each position and their trajectory. This analysis showed that there are no differences in the profile of cell cycle phases between cluster 1 and 2 cells (Supp. Figure 5h and Rebuttal Figure 3A below). We also did not find any difference in the cell cycle profiles of cells with oscillatory or non-oscillatory Venus::HES5 within the populations of clusters1 and 2 (Rebuttal Figure 3B below). These results are not surprising and they agree with our proposal that the noisy and oscillatory states are not static sub-populations but dynamic, plastic, outputs of the essentially the same gene expression system.

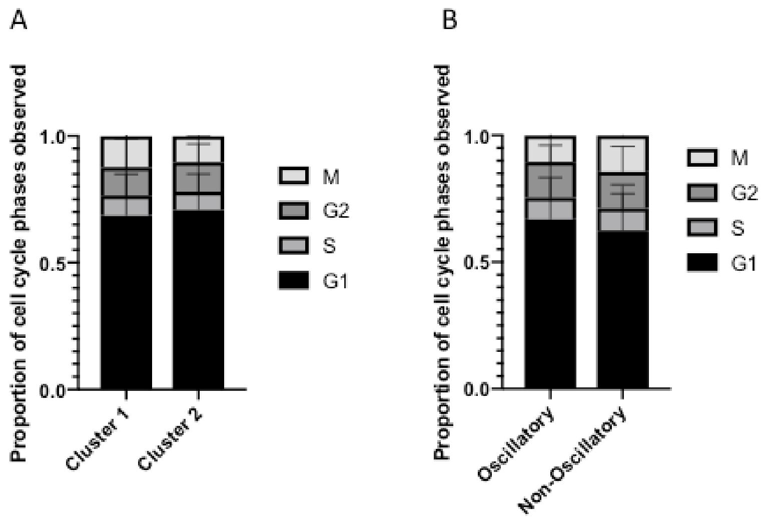

Figure 2. Cell cycle profiles are similar between different Venus::HES5 dynamics. Cell cycle profiles of A) cells in cluster 1 and 2 and B). Cluster 1 and 2 cells with oscillatory or non-oscillatory Venus::HES5.

3. Related to the above comment (#2), the functional significance of different Hes5 expression patterns in differentiating cells (clusters 3 and 4) is not clear. Is there any difference in differentiating cell types between oscillatory and non-oscillatory, or clusters 3 and 4?

Thank you for this comment that prompted us to look closely at whether such a difference exists and which ended up being a key finding in the revised version. We analysed the position of oscillatory and non-oscillatory differentiating cells in clusters 3 and 4, taking advantage of the fact that different neuronal sub-types are generated in different positions of the dorsal-ventral axis of the spinal cord. Combining the distance of cells from the floorplate and staining of the cultured ex-vivo slices for motor neuron and interneuron markers, we found that there is a higher incidence of oscillatory cells in differentiating cells that give rise to interneurons than in those giving rise to motorneurons. These results are summarized below (Rebuttal Figure 4) and are fully explained in Figure 6a-c and on page 12 of the revised manuscript. Thus, there are two paths by which HES5 declines, one of which is oscillatory and one which is not, and this correlates well with the fate that these cells adopt.

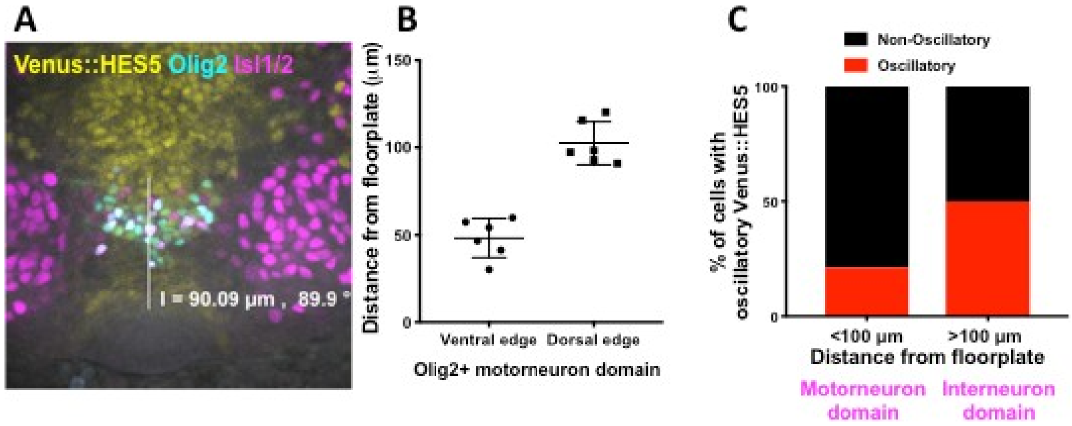

Figure 3. Oscillatory Venus::HES5 dynamics correlate with generation of interneuron fate. A). Immunohistochemical staining for the motorneuron progenitor marker Olig2 and motorneuron marker Isl1/2 in Venus::HES5 slice culture. B). Measurements of the ventral and dorsal edge of the motorneuron domain from immunohistochemistry of slice cultures. n=6 slices. C). Percentage of cells with oscillatory Venus::HES5 in domains less than 100 µm and greater than 100 µm from floorplate. 3 experiments combined.

4. Regarding the live imaging method, the authors should describe how many images with what depth intervals along the z axis were taken at each time point. Another issue is that in Supplementary Figure 2d, Venus-Hes5 signals in homozygous are not twice as high as heterozygous samples, suggesting that substantial background fluorescence may be included. The authors should remove the background fluorescence to quantify Venus-Hes5 signals.

Live imaging was performed using a Zeiss LSM880 microscope and GaAsP detectors with a Plan-Apochromat 20x 0.8 NA objective with a pinhole of 5AU. 10 z-sections were acquired every 7.5 µm every 15 mins for 18hrs. We have updated the methods to include this.

Background fluorescence (as measured via an ROI drawn outside of the cells) was subtracted prior to analysing time-lapse data and for image intensity quantification and this has been added to materials and methods for clarification.

The Venus::HES5 concentration in heterozygous and homozygous embryos was measured by fluorescence correlation spectroscopy (FCS). To show that tissue autofluorescence did not affect quantification, we have included FCS data collected in wild-type tissue (Rebuttal Figure 5 and Supp. Fig. 1h). As can be seen, WT tissue fluorescence count rate as measured by FCS was minimal (around 100x less) compared to true Venus::HES5 signal indicating that FCS measurements and quantification are unaffected by background.

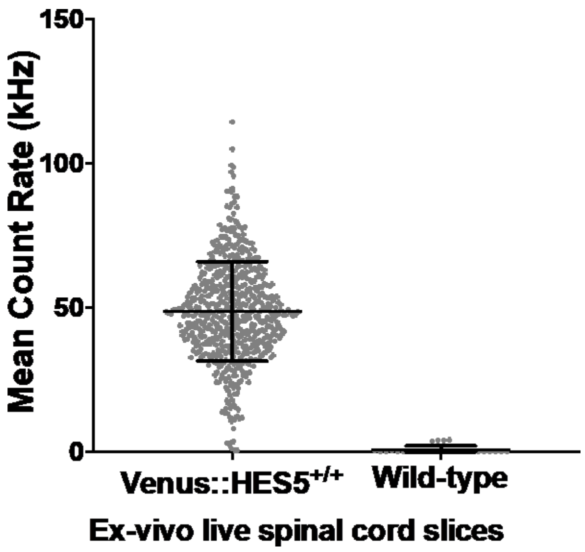

Figure 4. Fluorescence Correlation spectroscopy mean count rate per cell. Mean FCS count rate per cell from Venus::HES5 homozygous and Wild-type E10.5 ex-vivo slices.

--
Reviewer #2 (Remarks to the Author):

In this study by the Papalopulu lab, the authors develop quantitative approaches coupled with live imaging of a Venus::Hes5 reporter to describe the kinetics of HES5 protein expression in the mouse developing spinal cord. HES5 is a canonical target of the Notch pathway and acts as a repressor of pro-neuronal genes to maintain a progenitor state. Prior studies have shown that targets of the Notch pathway can display oscillatory expression in neural progenitors. These published studies indicate that Notch oscillations promote progenitor maintenance, while sustained expression promotes differentiation. In contrast, one key point of the current manuscript is that both progenitors and differentiating cells can transition between oscillatory and non-oscillatory states for HES5 expression. Further, using non-supervised hierarchical clustering of expression profiles, the authors show that putative differentiating cells are more likely to exhibit HES5 oscillations than putative progenitors. The authors conclude that in differentiating cells, increased likelihood of oscillations is associated with decreased HES5 expression, which coupled together may have a potent impact upon downstream target genes. Finally, using mathematical modeling based on measured Hes5 mRNA and protein turnover, the authors make the compelling point that oscillations arise from noise.
This is a novel and well written study. Using unbiased quantification of gene expression in tissue, this study illuminates fundamental, overlooked and therefore undocumented characteristics of oscillatory gene expression associated with neural differentiation in vivo. It relies upon elegant data using state of the art techniques. Given the potential discordance with previous data from the literature, some additional experiments are needed to support the authors’ claims. If the authors can accomplish these experiments, this study will

be valuable to the fields of neural differentiation and cell fate decisions at large.

Major concerns

The observation of differential oscillatory expression within progenitors vs differentiating cells relies on indirect clues about cell fate, such as relative distance from the ventricle, distance traveled, and presence of dividing cells. While these are valuable, a direct method is needed to verify the fate of cells as progenitors and neurons, such as using a Dcx-DsRed or Sox2-GFP transgene or immunofluorescence analyses following live-imaging. The authors need to show that oscillations occur in progenitors definitively and that it changes as cells differentiate.

We have tried to do this, but unfortunately, neither Dcx-DsRed (or another neuronal marker such as Tau:GFP, see below Rebuttal Figure 6) nor Sox2-GFP can be used for live imaging here, because they can not be resolved from the Venus::HES5 and the H2B::mCherry signals, both of which are necessary for the imaging experiment. We did in fact try combining Venus::HES5 with the Tau:GFP reporter (Mapt(tm1-EGFP) mouse) but as can seen from Rebuttal Figure 6, there is insufficient separation of GFP from Venus. One can subtract the GFP from the Venus signal (Rebuttal Figure 6C) but we would be very likely to lose some of the dynamics. Further the required narrow windows for emission wavelength resulted in very low signal when the tissue was cultured on the inserts required for long-term imaging (Rebuttal Figure 6D). The timing of onset of tau:GFP also limits its use for imaging the transition period. We have compensated for these problems by analyzing the cells by several different characteristics, namely the position, direction of travel, distance of travel and division of the cell and we feel therefore that it is quite robust in assigning cell state to dynamics. We would also like to point out that we have indeed used immunofluorescence for SOX2 and NeuN in relation to the Venus::HES5 expressing cells (Figure 1b and Figures 3a,b). This was performed in E10.5 spinal cord slices cultured ex-vivo and measurement of the SOX2-NeuN boundary in multiple slices allowed us to draw a mean boundary between the ventricular zone and mantle zone in figures 3a and b. The staining showed that the main HES5 expression zone coincides with SOX2 positive cells and that HES5 declines at the edge of the expression domain as it encroaches into the NeuN expression zone. This is entirely consistent with the rest of our interpretation.
Finally, please note that there is no discordance with previous data as the dynamics of Venus::HES5 have not been examined at a population level or during cell state transitions.

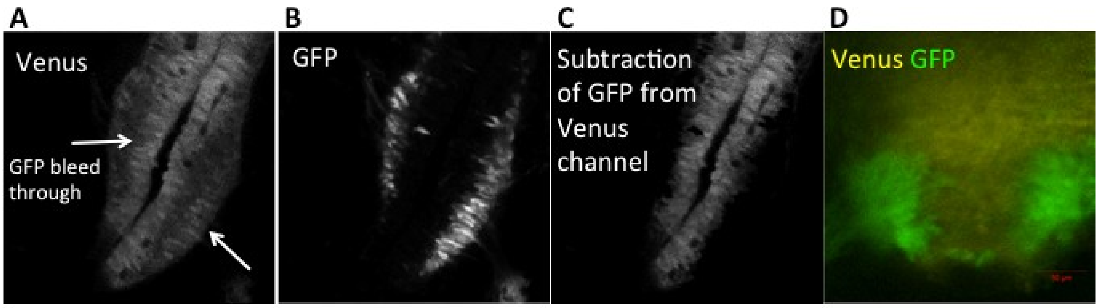

Figure 5. Tau:GFP reporter is difficult to spectrally separate from Venus::HES5 expression. Mapt(tm-GFP) mouse expresses GFP from Tau locus and marks mature neurons. E10.5 spinal cord slice from Mapt(tm1-EGFP) x Venus::HES5 mating placed directly on glass for imaging A). Venus channel B). GFP channel C). GFP signal subtracted from Venus signal. D). E10.5 spinal cord slice from Mapt(tm1-EGFP) x Venus::HES5 mating placed on cell-culture insert as used for long-term imaging. Poor signal in Venus channel due to narrow emission window.

It would also be valuable if the authors can use a genetic means to induce more progenitors and show that they all behave like clusters 1 and 2, ie. the reciprocal experiment to DBZ treatment.

Unfortunately, we do not have the genetic means to do this experiment. An older publication (Ohtsuka et al., 1999) used a lentiviral construct with constitutive active Notch (caNotch) to show that caNotch inhibits neuronal differentiation and the decline of HES5 (consistent with our interpretation that cells where HES5 declines are on their way to differentiation). We don't feel that lentiviral overexpression of this construct, which is also not suitable for live imaging, has the precision needed for our experiments. Although this experiment would have been nice, it would only be confirmatory in nature.

Distance from the ventricle could also correlate with cell cycle phase in progenitors. Thymidine analog-based analyses could be performed in order to interrogate this question.

Yes, indeed cell-cycle phase correlates with distance from the ventricle. We have been to estimate the cell-cycle phase in cells of cluster 1 and 2. Cells undergo INM, whereby nuclei undergo S phase further from the ventricle and return to the ventricle for M phase, separated by G1 and G2 phases. Indeed, we show an example cluster 1 cell in Fig.3a of the manuscript that undergoes inter-kinetic nuclear migration and migrates to the ventricular surface to divide. We have also added the apico-basal position of divisions in Supplementary Figure 5f and Rebuttal Figure 7A. In addition, we have calculated the cell cycle phases, based on how long nuclei spend in apical vs basal positions and their trajectory. This analysis showed that there are no differences in the profile of cell cycle phases

between cluster 1 and 2 cells (Supp.Fig 4g and Rebuttal Figure 7B). We also did not find any difference in the cell cycle profiles of cells with oscillatory or non-oscillatory Venus::HES5 within the populations of clusters1 and 2 (Rebuttal Figure 7C). These results are not surprising and they agree with our proposal that the noisy and oscillatory states are not static sub-populations but dynamic, plastic, outputs of the essentially the same gene expression system (added in revised manuscript page 13).

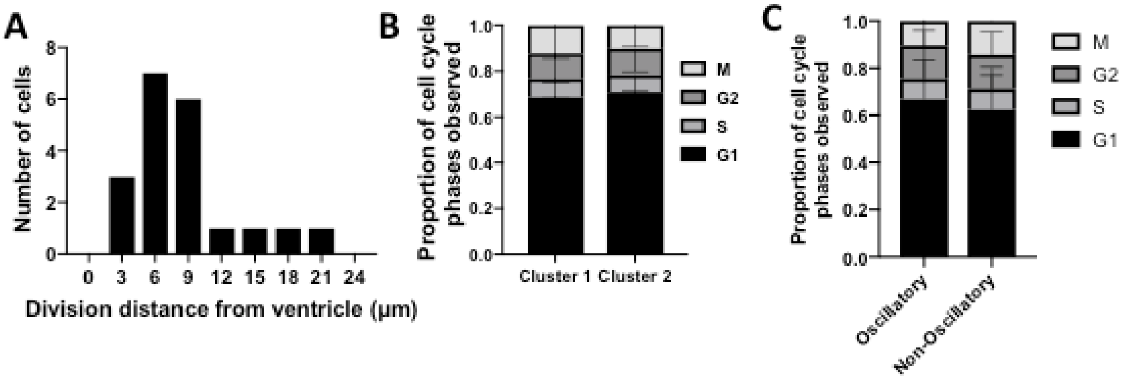

Figure 6. Divisions and cell cycle phase distribution in cells of cluster 1 and 2. A). Position of divisions in relation to the ventricle. Cell cycle profiles of B). cells in cluster 1 and 2. and C). Cluster 1 and 2 cells with oscillatory or non-oscillatory Venus::HES5.

I also have some questions regarding classification of cells. The classification of cluster 3 as differentiating cells is clear from the data included in Figure 3. However the cells of cluster 4 have a similar number of dividing cells as cluster 2 (Figure 3G) and exhibit similar distances from the ventricle (Figures 3C and F).

The reviewer is correct in pointing out cluster 2 and 4 have similar positions relative to the ventricle, however cluster 4 (14%) has fewer dividing cells than cluster 2 (21%). Further the trajectories of cells in cluster 2 and cluster 4 arevery different. Cluster 4 cells move basally during the movie, away from the ventricle, whereas cluster 2 cells have similar start and finish positions on average (see below Rebuttal Figure 8 and Fig. 3e in manuscript). The displacement of cluster 4 cells away from the ventricle is characteristic of differentiating cells and furthermore, some cluster 4 cells cross the boundary between the SOX2-positive ventricular zone and NeuN-positive mantle zone as determined by staining of slice cultures (Figure 3a,b in manuscript). The behaviour of cluster 2 cells is consistent with INM. We suggest that this is supports our idea of using multi-dimensional analysis to infer cell-state from multiple cell behaviors and tissue immunostaining.

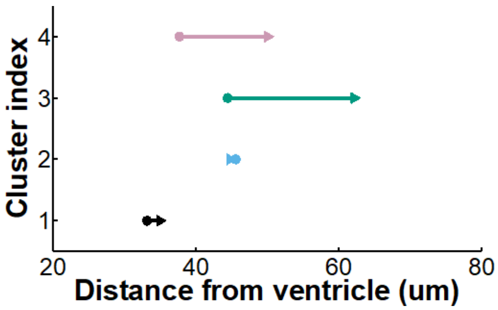

Figure 7. Trajectories of cells per cluster. Dots show average start position, arrowheads show average end position of 12 hr tracks from all experiments combined.

We note that Venus::HES5 mean intensity in cluster 4 starts to decrease later than cluster 3 (Figure 2f) thus, one can postulate that clusters 3 and 4 represent parts of the differentiation continuum with cells in cluster 3 representing later timepoints in differentiation than cluster 4. We reason that this is why cluster 4 shows more similarities to the progenitor clusters in some measures, such as division.

New analysis shows that clusters 1 and 2 also have a difference in expression level such that cells in cluster 1 have higher levels of Venus::HES5 than cells in cluster 2 when you normalize for distance into the tissue (Rebuttal Figure 9, Supp. Fig. 4h). But we not certain at the moment what this means and how cluster 1 is related to cluster 2.

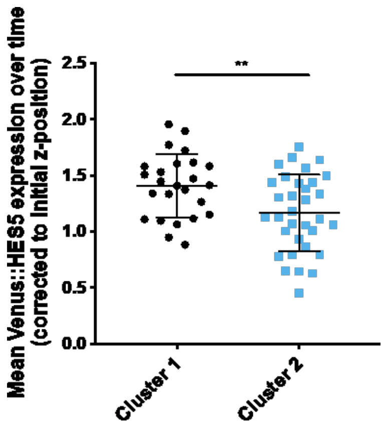

Figure 8. Venus::HES5 intensity is higher in cluster 1 cells than cluster 2 cells. Venus::HES5 intensity has been corrected for increased light scattering with increasing depth in the tissue by correcting to the initial z-position of the cell in the tissue.

To conclude, the simplest interpretation of our data is that clusters 1 and 2 give rise to clusters 4 and 3, representing transitions over time. To reflect the uncertainty of the relationship between clusters 1 and 2 or 3 and 4, we propose that presently it is more appropriate to think of classes 1 and 2 as one category and clusters 3 and 4 as another. Indeed, in the paper we sum up the dynamics of clusters 1+2 and 3+4 together (Figure 4d), We recognize that more complex alternatives, such as subtle heterogeneity in progenitors translated linearly to neuronal progeny heterogeneity, may exist and we state this explicitly in the

revised manuscript (page 9). Our paper represents the first step in resolving this type of dynamic heterogeneity.

The Notch inhibition experiments result in cells with patterns that are most similar to cluster 3 (comparing Figures 2E and 3K) however the authors conclude that DBZ treatment induces cluster 4 cells. How do the authors reconcile this and is it possible these clusters are overly simplified? Further characterization of these so-called DBZ induced differentiated cells is needed. For example, with the DBZ treatment do the authors see a similar fraction of dividing cells as in Cluster 4 of Figure 2?

We have looked at this again, and we believe that our original classification of most of the DBZ treated cells into cluster 4 versus cluster 3 is correct. Here is the reasoning :
Criteria for cluster assignment (as described in methods)
1. Mean Venus::HES5 intensity over time
Cells that are in cluster 4 tend to be a little delayed in the decrease in Venus::HES5 compared to cluster 3 cells in untreated (Fig. 2f) or DMSO treated tissue (Rebuttal Fig. 10.A)
2. Coefficient of variation (C.O.V) of Venus::HES5 over time
Cluster 3 cells have the greatest C.O.V of Venus::HES5 over time, and cluster 4 the next highest (Fig. 3b), Rebuttal Figure 10.B).

These criteria are upheld in DBZ treated tissue. See Rebuttal Fig. 10.A below for mean Venus::HES5 intensity over time in DBZ vs DMSO treated tissue. Rebuttal Fig. 10.B shows C.O.V of Venus::HES5 over time for untreated tissue all cells combined and C for DBZ treated tissue. Please also find these plots in Supp. Fig. 5c,d.

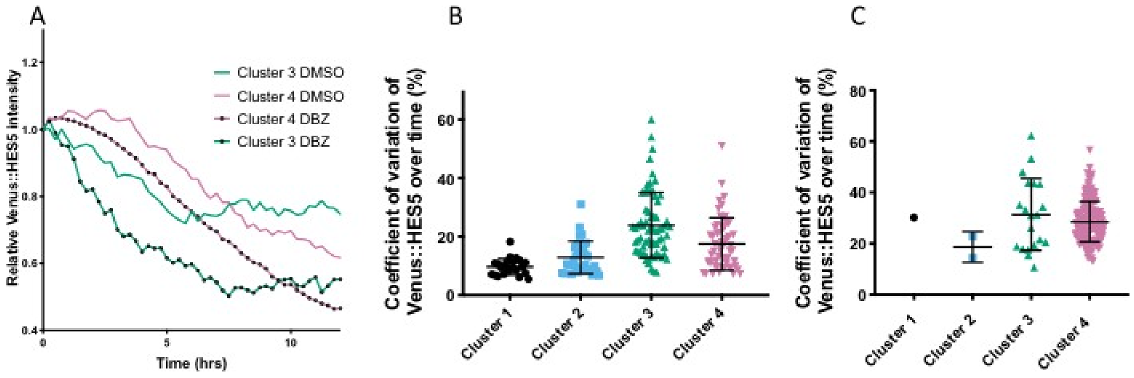

Figure 9. Cluster identification and annotation of cells in DMSO and DBZ treated ex-vivo slices. A). Mean Venus::HES5 expression dynamics for cells in each cluster in DMSO and 2μM DBZ treated ex vivo slices. B) Coefficient of variation of Venus::HES5 expression in a single cell

over time in untreated ex-vivo slices. All experiments combined. C) Coefficient of variation of Venus::HES5 expression in a single cell over time in DBZ treated ex-vivo slices.

We looked at the number of divisions in cluster 3 and 4 in DBZ treated tissue as suggested. We find that in DBZ treated tissue cluster 4 has a higher proportion of divisions than cluster 3 as also seen in the untreated tissue data in figure 3g.

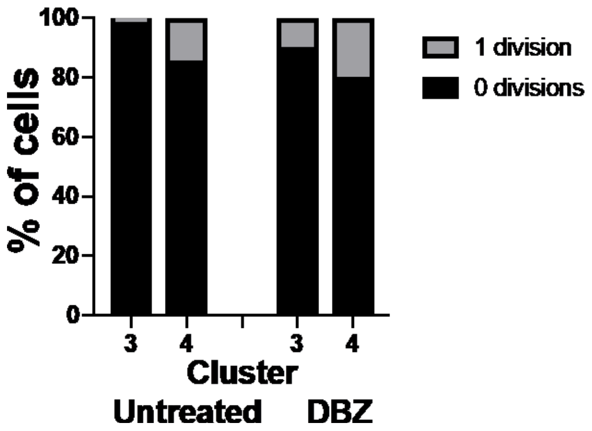

Figure 10. Dividing cells in cluster 3 & 4 in DBZ treated ex-vivo slices. Percentage of cells showing a division in 12 hour track in untreated and 2µM DBZ treated ex vivo slices.

Therefore the timing of the decline, the COV of Venus::HES5 over time and the percentage of dividing cells is more consistent with most of the DBZ cells falling into cluster 4-type dynamics. As we mentioned in our answer to a previous point, it is not clear at this point how cluster 3 and 4 are related to each other, although the simplest interpretation is that in normal development cluster 4 cells are at an earlier stage of the declining trajectory than cluster 3.

Minor points

On page 7 they refer to dynamic fluctuations represented by scenario Fig. 2a ii but it seems these should refer to Fig. 2a i.

Thank you for pointing this out, we have changed this in the manuscript

Do the authors observe any influence of local neighbor on fluctuations or coordination of oscillations amongst neighboring cells, suggesting a non-cell intrinsic mechanism at play?

Thank you for this comment; we agree that it is a highly interesting point. Due to the mosaic labeling of H2B::mCherry in SOX1+ neural progeny we are unable to look at closely neighbouring cells in this dataset. The changes in the number of oscillations that we observe in dissociated cells (added in Supp. Fig. 8a) argues in favour of a non-cell autonomous effect in regulating the dynamics. However, the influence of neighbouring cells and the spatial co-ordination of oscillations

and fluctuations between cells in the tissue is currently the focus of another project in the lab and we feel that it is outside of the scope of this work.

In figure 1 they monitor Sox1-Cre recombined cells after several hours, which they refer to as neural progenitors, but these could be neuronal progeny. This point should be modified.
Yes, we agree. The tamoxifen to induce recombination of H2BmCherry in SOX1-positive cells was administered 18 hours before imaging, and so labels cells that were SOX1-positive in the last 18hrs. Thank you for this comment, we have altered this where it appears in the text.

--

Reviewer #3 (Remarks to the Author):

The manuscript by Manning et al describes an experimental and computational analysis of single cell Hes5 dynamics in the developing mouse spinal cord. The authors use quantitative live imaging of Venus:Hes5 knock-in reporter to look at both the mean fluorescence and the fluctuations around the mean of Hes5 levels in neural progenitors in the spinal cord. The authors identify 4 groups of cells exhibiting different dynamics of the mean fluorescence, where 2 groups (group 1 and 2) correspond to generally constant levels of Hes5 and the two other groups correspond to decay in fluorescence over time (groups 3 and 4). They then show that two latter groups are mostly associated with cells transitioning into a differentiated. The authors then argue that some of the cells exhibit periodic oscillations around the mean (as opposed to aperiodic fluctuations), and that these periodic oscillations occur predominantly the decay in fluorescence in groups 3 and 4. The authors use a stochastic
delayed feedback model to explain the observed cellular dynamics. They argue that the periodic fluctuations may be important for the transitions into a differentiated state.
Although the issue of cellular dynamics is indeed an important topic, and although the authors do a very detailed and careful analysis of the Hes5 single cell dynamics, I unfortunately do not find the study to be very compelling, nor the results (particularly those describing the oscillations) very convincing.

The main issue is that unlike previous studies in neural progenitors in the brain, the oscillations reported here are very weak (namely, have very low amplitude about the mean). Although I appreciate the quantitative effort to show that a periodic model fits better some of the curves than aperiodic fluctuating model, I am still skeptical whether the observed fluctuations are real oscillations or not (see more detailed concerns below).

We understand the reviewer's concern. However, the notion of a perfect HES oscillator with a defined periodicity and a large amplitude is a misconception, propagated in review diagrams. As we said above (see reviewer 1) previous analysis of HES5 focused on a very limited number of cells, which were

dissociated and cultured in vitro and had no statistical analysis for the presence of oscillations. There was no characterisation of periodicity or amplitude. The paper of Imayoshi et al. 2013 does indeed show a couple of cells with large amplitude, but these are driven by a reporter fragment and they are in dissociated cells, not in a tissue environment. Here, we have undertaken a description of dynamics at a population level (but with single cell resolution), backed up by statistical analysis; a characterisation of the periodicity and amplitude is reported for the first time in our paper. We have invested a lot of time in developing a statistical method that would be suitable for our data and this has been peer reviewed and published in PLOS Comp. Biology(Phillips et al., 2017) (and an improvement for fluorescent data is included in the present manuscript). We believe that these are major strengths of our work and our findings represent the biology of the system.

Furthermore, as we also said in response to the comments of reviewer 1, we propose that the functional significance of the dynamic expression in progenitor cells is to keep the expression activated but plastic, that is, poised to undergo a transition. This hypothesis is borne out by experimental observations that show that only less than half of the cells oscillate and our computational experimentation which shows that the system is poised at a bifurcation point. It is unlikely that the oscillations in progenitors are decoded as such, because their amplitude is small (Maximal peak:trough fold-change of 1.25x on average in cluster 1, 1.37x in cluster 2, Figure 6a). We have re-written this part to make it clearer. However, in clusters 3 and 4 the oscillatory amplitude is combined with a downward trend, to generate higher instantaneous fold changes (Figure 6a,b,c,d). This is likely to be the decoding phase of the oscillator. Indeed, in the revised manuscript we provide evidence that oscillations or monotonic decline correlate with different neuronal populations (Fig.6g,h,i), suggesting that oscillations are decoded during the route to differentiation

Furthermore, even if these oscillations are real, it is unclear to me whether they represent an oscillating state of the cell. To really show that the cells have an oscillating state, the authors should have a two color reporter in two loci of the same gene or of two separate genes that show correlated oscillations. I realize this is a big task experimentally, but I think that the authors would have hard time to convince the community that these are oscillatory states without such evidence.

Producing a 2–color reporter by genetically engineering single copy knock-ins in the 2 *Hes5* loci is outside what we can achieve within the time constraints of this study. However, we understand the point that the reviewer makes and we have found another way to address is. We believe that the reviewer essentially asks whether the cell “experiences” oscillations in total Venus::HES5 levels (which he/she calls an “oscillatory state”). To address this question we have performed new experiments to compare the presence of oscillations in dissociated neural

progenitor cells from the spinal cord of mice that are heterozygous with those that are homozygous. If the reviewer's concern was true, we would not find oscillations in cells from the homozygous mice. However, our data showed that there is no difference in the percentage of oscillatory cells in heterozygous or homozygous reporter mice (Rebuttal Figure 12 and Supp. Fig.8a). This finding not only addresses the reviewer's legitimate concern, but provides some deeper mechanistic insight. Namely, this finding means that HES5 oscillations, driven by transcriptional auto-inhibition, are quite distinct from stochastic transcriptional bursting. It is well known that the latter is averaged out to a flat line when transcription for the 2 loci is measured because transcription from the 2 loci is uncorrelated. We discuss this point on page 14 of the revised manuscript. We think this is a very nice addition to the revised manuscript and we thank the reviewer for making this point.

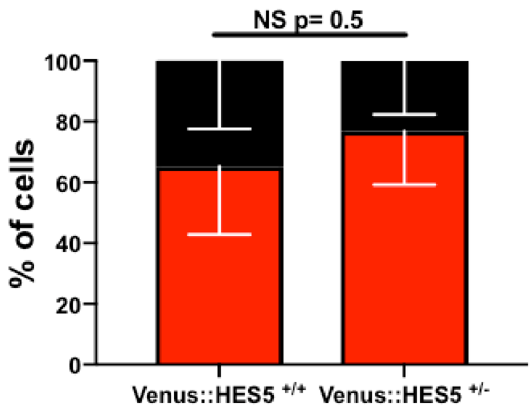

Figure 11. Comparison of percentage of oscillators in heterogygous vs homozygous cells. Percentage of cells with oscillatory Venus::HES5 expression dynamics in dissociated primary neural stem cells from spinal cord of Venus::HES5 homozygous vs heterozygous embryos.

In addition to this main criticism there are several more specific points:
1. The authors show that cells in groups 3 and 4 exhibit a decays in the signal and that this decay correlate with exit from the ventricular zone to the mantle zone. Do any of the H2B:mCherry cells also exhibit such a decay? Is it correlated with distance away from the ventricle?

The H2B:mCherry cells do not show decay or oscillations, neither close nor far away from the ventricle. We have done additional testing in the revised manuscript (see Rebuttal Figure 13 and added in Supp. Figs 3c,4c,e).

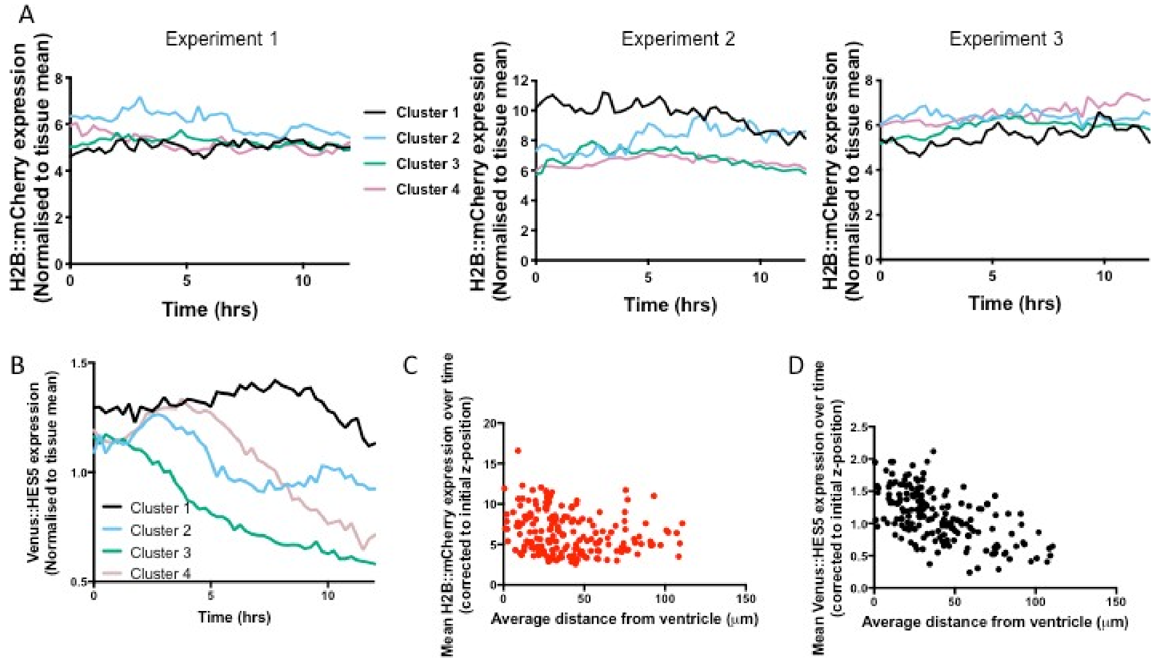

Figure 12. H2B::Cherry dynamics are similar between clusters and does not decrease with increasing distance from ventricle. A. Mean H2B::mCherry expression dynamics for cells in each cluster in each experiment. B. Mean Venus::HES5 expression dynamics for cells in each cluster in representative experiment. C. Average distance from the ventricle over 12 hr track vs mean H2B::mCherry corrected intensity. D. Average distance from the ventricle over 12 hr track vs mean Venus::HES5 corrected intensity. Intensity was corrected for the increased light scattering at increasing depth (z-position) in to tissue by normalising to initial z-position in tissue.

2. The oscillating are predominantly observed in groups 3 and 4 during the decay. Is there a correlation between mean Hes5 levels and the oscillatory behaviors? Namely, could it be that the observed oscillatory behavior appears when the signal gets weaker?

We can see how one would perhaps expect that lower protein level may make oscillations appear. To address this, first, we plotted the likelihood of being oscillatory (LLR score) versus the Venus: HES5 intensity values (Rebuttal Figure 14A and Supp. Fig.10b). Second, we plotted the mean Venus:Hes5 level over time in oscillatory and non-oscillatory cells. We analysed all cells together (forthe reviewer only) in addition to just cluster 1&2 cells (Rebuttal Figures 14B,C and Supp. Fig.10a). In both cases the Venus::HES5 signal was first corrected for the depth in to the tissue at the first time point as increasing depth results in increased light scattering. The overarching conclusion from both types of analysis is that oscillatory behavior does not correlate with the mean level of expression. Putting it more simply, oscillations are not caused, as far as we can tell, by the level of expression. This additional data analysis is included in the revised version and discussed in the text on page 12.

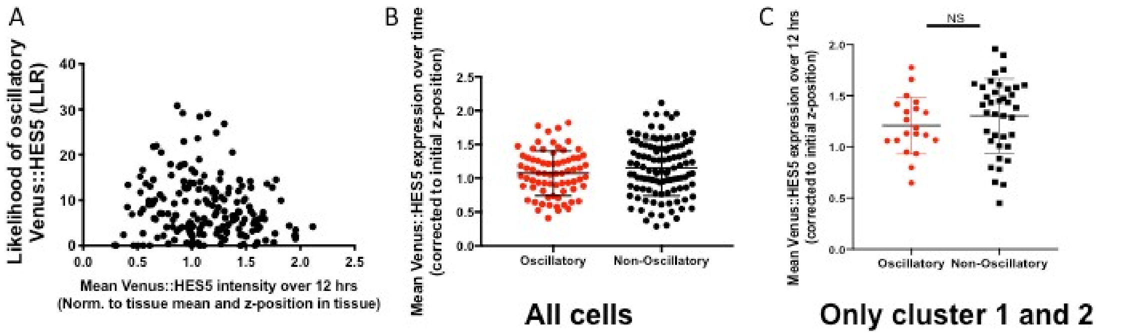

Figure 13. Levels of Venus::HES5 do not correlate with oscillatory dynamics. A) Mean Venus::HES5 intensity of cells over 12 hr track corrected for z-position in the tissue vs likelihood that cell has oscillatory Venus::HES5 (LLR from stochastic test). B) Mean Venus::HES5 intensity over 12 hr track corrected for z-position in the tissue in all cells with oscillatory or non-oscillatory Venus::HES5 expression. C) Mean Venus::HES5 intensity over 12 hr track corrected for z-position in the tissue in cells of cluster 1 & 2 with oscillatory or non-oscillatory Venus::HES5 expression. NS is no significance in Student t-test.

3. Are the oscillations also observed when DBZ is added? If so, is there a larger fraction of cells exhibiting oscillations with DBZ (one would predict that based on the observation that oscillations appear when Hes5 signal decays).

In the revised version, we have analysed DBZ treated slices for the proportion of oscillatory cells. The proportion of cells with oscillatory Venus::HES5 is slightly greater when treating with DBZ versus the DMSO control, but this is not significant. There are also no significant differences when compared to only cells in cluster 3 and 4 in DMSO treated ex-vivo slices (Rebuttal Figure 15 and Supp. Fig. 10d). Therefore in DBZ conditions, which lead to differentiation, there are as many oscillators as you would expect to find in the differentiating cells of control tissue. This finding suggests that the oscillations are not caused by the signal decay but by some other mechanism, perhaps involving interactions with other genes, which takes place at the same time that the level decreases. The new data are shown in Supp.Fig. 10d and mentioned on page 12 of the revised manuscript.

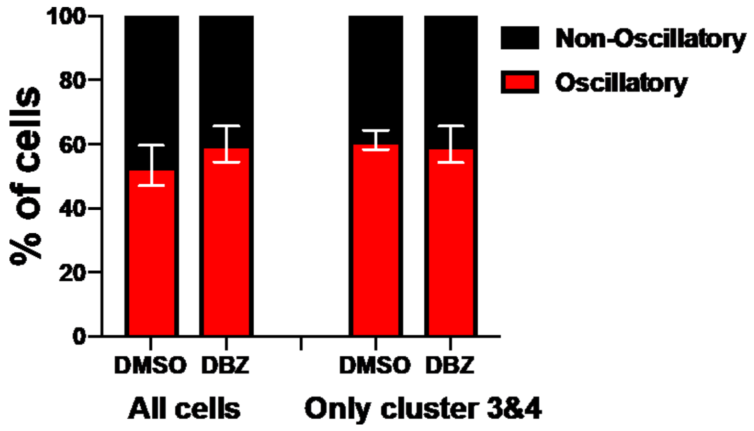

Figure 14. No difference in incidence of oscillatory Venus::HES5 in DMSO vs DBZ treated ex-vivo slices. Percentage of cells with oscillatory Venus::HES5 expression dynamics in heterozygous ex-vivo spinal cord slices treated with DMSO or 2μM DBZ, showing all cells or just cells of cluster 3&4. NS - No significance in Student t-test.

4. In the paragraph discussing figure 4 (page 9) it is said that 47% of the cells exhibit oscillatory behavior. However, in fig 4c the fraction of oscillating cells seem to be lower in almost all groups tested. Why is there such a discrepancy?

Thank you for pointing this out, it was a mistake and we have corrected it to read that 41% of cells exhibit oscillatory behaviour.

5. Figure 4e shows that clusters 1 and 2 have higher noise than clusters 3 and 4. Could this simply reflect the higher mean levels in these groups (note that the CV of clusters 1 and 2 are lower as show in in fig. 2h).

We have looked at this and we find that, unlike oscillations (point 2 above) there is a positive correlation between the amount of noise and the mean Venus-HES5 expression (Rebuttal Figure 16 and Supp. Fig 10c). This is in fact exactly what one would expect, based on the known propensity of noise to scale with the mean level. Thank you for prompting us to look into these points with more detail, as the results supported our findings and strengthened the manuscript.

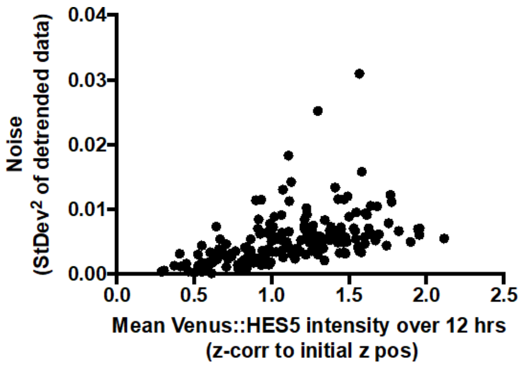

Figure 15. Noise in Venus::HES5 dynamic expression correlates with level. Mean Venus::HES5 intensity over 12 hour track corrected to z-depth of cell in tissue vs noise in Venus::HES5 expression. Noise is measure as the squared standard deviation of the de-trended Venus::HES5 expression over the 12 hour track.

6. In figure 4f likelihood of oscillatory behavior is shown to be higher for cells away from the ventricle. Could the authors also show how the likelihood depend on absolute mean level of Hes5?

This question has overlap with point 2 above and we kindly refer the reviewer to our reply above. In summary, we did not find a correlation between the likelihood to oscillate and the mean level of expression, but we do find a correlation with the declining trend in expression.

7. Similarly, the authors should also show how the noise in Fig 4g depends on mean level.

Yes, we have done this as explained above (reply to point 5). There is a small positive correlation of noise with mean level, as one would expect.

8. The authors argue that the typical behavior is that oscillation appear only after the Hes5 signal starts to decay (Figure 4h and 6f). Can the authors determine what fraction of cells exhibit this type of typified behavior? For example, by calculating in what fraction of cells the oscillations appear on the second half of the measurements.

Technical limitations prevented us from providing this exact calculation. Our measurements are done in live tissue slices and the traces are not long enough to enable us to "split" them in temporal halves. The statistical method that enables us to detect oscillations loses power in short time-series, which is expected. We have characterised the "shoulder" point in the decay of the signal, i.e the start of the decrease in Venus::HES5 and its distance from the ventricle; this shown in Fig 4d.

9. I find the stochastic model to be not particularly illuminating. First, it is unclear why the model applies only to clusters 1 and 2 (first sentence in section 7 of the result). Second, it is not clear to me what is gained by the model. The fact that such a model CAN fit partially fluctuating and partially oscillating behaviors is not particularly surprising. Does the model generate a prediction that can be tested experimentally?

The reviewer posed some interesting questions that helped us improve the presentation of the computational part of the paper. To answer the first part, the model applies to clusters 1 and 2 because it specifically models the transition from noisy to oscillatory in progenitor cells. These cells oscillate around a flat

mean. In order to model the behavior in clusters 3 and 4, we would need to model the HES5 signal decay (in addition to the oscillatory behavior). At the moment, there is insufficient experimental evidence to construct a model that will represent the decay in HES5. There is some evidence that the HES5 signal decay in motor neurons is mediated by OLIG2 (Sagner et al., 2018) and we have discussed this in the paper, but it is not known what mediates the decay in interneurons. Therefore, this is work for the future that should involve experimentation as well as computation. We agree that these points needed clarification and we have done this in the text, pages 10 & 14.

To answer the second part, the model makes a number of important predictions, which we can outline here. First, the model predicts that changes in the protein stability and/or mRNA stability will affect the likelihood that HES5 will oscillate (Fig. 5e,f,g). Secondly, the model estimates distributions for the parameter values that explain the protein expression dynamics and can serve as predictions of their values. These are estimated by Bayesian Inference as “posterior probabilities” and are shown in Supp. Figure 9d. In new work we have explored the parameters that are most likely to induce oscillations with a period of less than 5 hrs from aperiodic fluctuations. The modeling predicts that an increase in the Hill coefficient, a reduction in the repression threshold, and an increase in protein degradation are the most likely parameter changes to induce oscillations (Rebuttal Figure 17). Please find these additions in Fig. 5h and text pages 11 & 13. These predictions are very important for guiding and prioritizing future experiments. This model is also a very useful starting point on which to model (in the future) the decay in the HES5 signal. Thank you for prompting us to be more specific about these points, as this improved the manuscript.

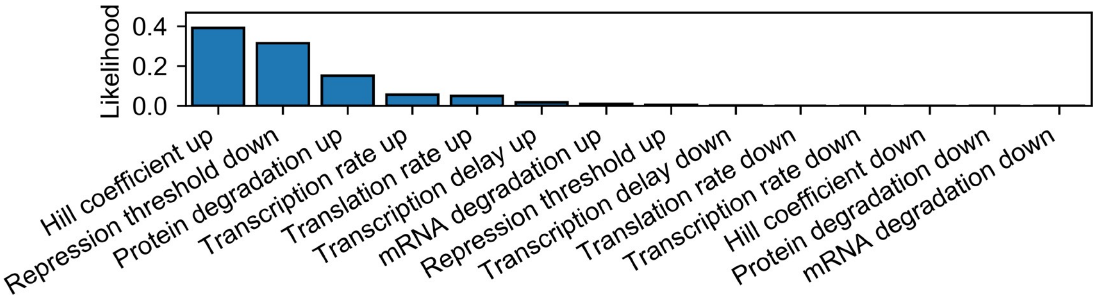

Figure 16. Likelihood of inducing oscillations with less than 5hr period from aperiodic fluctuations through changing individual parameters by 50%.

References

Ball, D. A., Lux, M. W., Adames, N. R. and Peccoud, J. (2014) ‘Adaptive Imaging Cytometry to Estimate Parameters of Gene Networks Models in Systems and Synthetic Biology’, *PLoS ONE*. Edited by M. Thattai. Public Library of Science, 9(9), p. e107087.

Imayoshi, I., Isomura, A., Harima, Y., Kawaguchi, K., Kori, H., Miyachi, H., Fujiwara, T., Ishidate, F. and Kageyama, R. (2013) ‘Oscillatory control of factors

determining multipotency and fate in mouse neural progenitors.', *Science*, 342(6163), pp. 1203–1208.
Kobayashi, T., Iwamoto, Y., Takashima, K., Isomura, A., Kosodo, Y., Kawakami, K., Nishioka, T., Kaibuchi, K. and Kageyama, R. (2015) 'Deubiquitinating enzymes regulate Hes1 stability and neuronal differentiation', *FEBS Journal*, 282(13), pp. 2411–2423.
Ohtsuka, T., Ishibashi, M., Gradwohl, G., Nakanishi, S., Guillemot, F. and Kageyama, R. (1999) 'Hes1 and Hes5 as notch effectors in mammalian neuronal differentiation.', *The EMBO journal*. EMBO Press, 18(8), pp. 2196–207.
Phillips, N. E., Manning, C., Papalopulu, N. and Rattray, M. (2017) 'Identifying stochastic oscillations in single-cell live imaging time series using Gaussian processes', *PLOS Computational Biology*. Edited by J. Listgarten. Public Library of Science, 13(5), p. e1005479.
Sagner, A., Gaber, Z. B., Delile, J., Kong, J. H., Rousso, D. L., Pearson, C. A., Weicksel, S. E., Melchionda, M., Mousavy Gharavy, S. N., *et al.* (2018) 'Olig2 and Hes regulatory dynamics during motor neuron differentiation revealed by single cell transcriptomics', *PLOS Biology*. Edited by M. Bronner. Public Library of Science, 16(2), p. e2003127.

REVIEWERS' COMMENTS:

Reviewer #1 (Remarks to the Author):

The authors have carefully addressed my concerns, and I have no further comments.

--
Reviewer #2 (Remarks to the Author):

This revised manuscript by Papalopulu and colleagues uses live imaging to measure Hes5 oscillations in presumed progenitors and neurons of the developing spinal cord. A major strength of this manuscript is the in vivo analysis and modeling, which previously had primarily been done in vitro. The authors have added extensive new data, including analyses of cell cycle state and correlative relevance of oscillations in differentiating interneurons versus motor neurons. While the paper is largely correlative/descriptive, it presents a piece of work that will enable researchers to generate new hypotheses about dynamic cellular states, evidenced by oscillating Hes5 gene expression, during development. Thus I feel it is much improved and my major concerns have been addressed.

Minor point
In several main figures the images and graphs should be adjusted for readibility. In some main and supplementary figures, graph fonts are disproportionately too small relative to other graphs or figures (for example, Figure 1c,d, Sup figure 2).

The authors demonstrate there are more oscillations in vitro versus in tissue, which they posit suggest tissue environment can affect HES5 dynamics (non-cell autonomous). However as this is comparing "apples and organges", another interpretation is the culture conditions used here are simiply biased towards more oscillations (or increased fraction of cells which exhibit oscillations). I think this important point needs to be mentioned in the discussion and possibly results (p. 9).

--
Reviewer #3 (Remarks to the Author):

In the revised version the authors have done significant improvements to the manuscript that address many of the questions raised by me and the other reviewers. In particular they provided evidence for potential correlation between the oscillatory state and the neuronal fate. They also provided additional evidence that the oscillations in Hes5 are not simply due locus specific transcriptional bursts. Finally, they have improved the presentation of the model to show the predictions. Although I am still not certain that the Hes5 oscillations are indeed functionally important (see below) I agree that the data and detailed analysis presented should be published and made available to the community.

There are a couple of issues that need to be addressed:

1. In the new data the authors show that the distance from the floor plate correlates with neuronal fate and also that the distance from the floor plate is correlated with the fraction of oscillatory cells. As far as I understand they use these to claim that oscillatory state correlates with neuronal subtype (see abstract). If this is indeed the claim, this is incorrect logic as correlation of A with B and correlation of A with C do not imply correlation between B and C. Therefore, the authors should find a way to directly correlate oscillatory state with neuronal fate to support their point.

2. In the new data provided the authors show that the mean expression does not affect the likelihood

of oscillations but does correlate with the fluctuations in clusters 1 and 2. This is actually an interesting behavior that should be captured by the mathematical model. The authors should therefore check if their model captures these behaviors. I believe this would strengthen the manuscript significantly.

3. A clarifying point: in my previous review I asked whether the oscillatory behavior corresponds to a real cell state. The authors provided stronger evidence for the presence of Hes5 oscillations. But the question remains whether multiple regulators oscillate together with Hes5 or whether each regulator oscillate independently. I believe that if the second case is found to be true, it would be harder to argue that the oscillations are really associated with a cell state. I realize that a two color experiment is beyond the scope here, but it might be useful to point out this question in the discussion.

Thank you for the reviews of our revised paper. We were pleased to see that all reviewers were happy with our revisions. We understand that there are some additional minor questions and we thank you for giving us the chance to reply to these questions. Here we address these issues on a point-by-point basis. We hope these revisions will allow you to accept our manuscript for publication.

REVIEWERS' COMMENTS:

Reviewer #1 (Remarks to the Author):

The authors have carefully addressed my concerns, and I have no further comments.
Thank you for your kind reviews of our paper.
--
Reviewer #2 (Remarks to the Author):

This revised manuscript by Papalopulu and colleagues uses live imaging to measure Hes5 oscillations in presumed progenitors and neurons of the developing spinal cord. A major strength of this manuscript is the in vivo analysis and modeling, which previously had primarily been done in vitro. The authors have added extensive new data, including analyses of cell cycle state and correlative relevance of oscillations in differentiating interneurons versus motor neurons. While the paper is largely correlative/descriptive, it presents a piece of work that will enable researchers to generate new hypotheses about dynamic cellular states, evidenced by oscillating Hes5 gene expression, during development. Thus I feel it is much improved and my major concerns have been addressed.
Thank you for your careful reviews of our paper. We are pleased that you feel it is improved.

Minor point
In several main figures the images and graphs should be adjusted for readibility. In some main and supplementary figures, graph fonts are disproportionately too small relative to other graphs or figures (for example, Figure 1c,d, Sup figure 2).
We have endeavoured to change the size and texts of graphs.
The authors demonstrate there are more oscillations in vitro versus in tissue, which they posit suggest tissue environment can affect HES5 dynamics (non-cell autonomous). However as this is comparing “apples and organges”, another interpretation is the culture conditions used here are simiply biased towards more oscillations (or increased fraction of cells which exhibit oscillations). I think this important point needs to be mentioned in the discussion and possibly results (p. 9).
We have changed the results to include
“…suggests that HES5 dynamics are influenced by the tissue environment, although many factors change between in vitro and ex vivo conditions.”

--

Reviewer #3 (Remarks to the Author):

In the revised version the authors have done significant improvements to the manuscript that address many of the questions raised by me and the other reviewers. In particular they provided evidence for potential correlation between the oscillatory state and the neuronal fate. They also provided additional evidence that the oscillations in Hes5 are not simply due locus specific transcriptional bursts. Finally, they have improved the presentation of the model to show the predictions. Although I am still not certain that the Hes5 oscillations are indeed functionally important (see below) I agree that the data and detailed analysis presented should be published and made available to the community.
Thank you for your thorough reviews of our paper. We are pleased that you feel it is a detailed analysis that should be published.
There are a couple of issues that need to be addressed:
1. In the new data the authors show that the distance from the floor plate correlates with neuronal fate and also that the distance from the floor plate is correlated with the fraction of oscillatory cells. As far as I understand they use these to claim that oscillatory state correlates with neuronal subtype (see abstract). If this is indeed the claim, this is incorrect logic as correlation of A with B and correlation of A with C do not imply correlation between B and C. Therefore, the authors should find a way to directly correlate oscillatory state with neuronal fate to support their point.
We can see the logic of the reviewer, however this is not the logic we use. We would like to clarify that distance from the floorplate does not merely correlate with neuronal sub-type, but distance from the floorplate specifically instructs neuronal sub-type. A significant body of work on spatial patterning of the spinal cord shows that a morphogen gradient in Shh arising from the floorplate generates clearly delineated domains that each give rise to different neuronal sub-types. Thereby at a certain distance from the floorplate, there is a known neuronal sub-type generated. We show that in the region where interneurons are generated there is a significantly higher proportion of cells with oscillatory Venus::HES5 compared to the domain where motor neurons are generated.

We have clarified the above in the text by saying that distance from the floorplate specifically instructs neuronal sub-type and we have edited the use of the term “correlate”.

2. In the new data provided the authors show that the mean expression does not affect the likelihood of oscillations but does correlate with the fluctuations in clusters 1 and 2. This is actually an interesting behavior that should be captured by the mathematical model. The authors should therefore check if their model captures these behaviors. I believe this would strengthen the manuscript significantly.
In the previously revised manuscript on page 12 we write
“As expected we did find a positive relationship between Venus::HES5 levels and noise (Supp. Fig.10c)”

As Venus::HES5 levels increase, the absolute variations in the aperiodic

fluctuations increases and the reviewer suggests this ought to be reflected in the model. Indeed, this observation is not novel and it is a well-known mathematical property of the Chemical Langevin Equations that we use in this paper (Gillespie, 2000; Van Kampen, 2007). Hence the reviewer's hypothesis that this could be a property of the model is correct. To clarify this we have changed the sentence quoted above and add references;

"As expected (Gillespie, 2000; Van Kampen, 2007) we did find a positive relationship between Venus::HES5 levels and noise (represented by absolute variance) (Supp. Fig.10c)"

To show this explicitly in our model, we have changed the repression threshold, which leads to changes in mean Venus::HES5 levels. This allows us to make a Bayesian Posterior prediction for the relationship between mean and variance of HES5 expression. We find that indeed, simulations with higher mean Venus::HES5 levels have increased variance (Fig.1), indicating that the model does capture the correlations observed between mean levels and noise observed in the experimental data. We have added this to Supplementary Figure

Figure 1. Increasing variance with increasing Venus::HES5 mean is

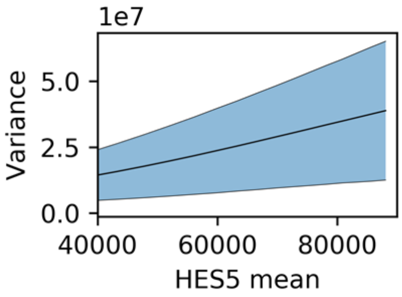

captured by the model.

We have also specifically outlined which behaviours in the data are yet to be captured by the model. We have inserted the following text to the discussion (end of paragraph 3, page 13).

"Future development of this model will capture remaining features of the observed dynamics such as the down-regulation observed during the differentiation process, as observed in cells of cluster 3 and 4, as well as other gene regulatory interactions and multi-cellular interactions."

3. A clarifying point: in my previous review I asked whether the oscillatory behavior corresponds to a real cell state. The authors provided stronger evidence for the presence of Hes5 oscillations. But the question remains whether multiple regulators oscillate together with Hes5 or whether each regulator oscillate independently. I believe that if the second case is found to be true, it would be harder to argue that the oscillations are really associated with a cell state. I realize that a two color experiment is beyond the scope here, but it might be useful to point out this question in the discussion.

We would like to clarify that we use “oscillating state” to mean that Venus::HES5 is oscillatory and not that all or multiple genes in the cell are oscillatory. We have clarified this, especially in the discussion. We agree that the question of whether HES5 oscillates in-phase or out-of-phase with other potential oscillatory genes in the cell is interesting and remains to be addressed. We have addressed this by adding the following paragraph to the discussion (page 15).

“Other genes in the Notch-Delta network such as Hes1, Dll1 and Ngn2 have been shown to oscillate in neural progenitors. The relative timing of pulses of different genes may regulate cellular behaviour as common target genes may respond differently to in-phase or out-of-phase input pulses. Indeed, the relative phase of the Notch and Wnt signalling oscillations in somitogenesis have been proposed to control cellular differentiation. Imaging protein expression dynamics of multiple factors in the same cell during cell fate decisions would help to reveal the relative timing of multiplexed oscillatory gene expression”.

References:

Gillespie, D.T. (2000). The chemical Langevin equation. J. Chem. Phys. *113*, 297–306.
Van Kampen, N.G. (2007). Stochastic Processes in Physics and Chemistry (Elsevier).
